# Spectacles

**Published:** 1874-11

**Authors:** 


### SPECTACLES.

This is the name given to glasses which assist the eye in seeing better, when they are attached to the head; "eye-glasses," when they are worn without any such attachment, to be held by a string or carried in the vest pocket.

Spectacles become necessary when you first notice yourself going to the window, instinctively, for a better light. You next find yourself moving the paper farther from your eyes than usual; about the same time you oftener lay it down and begin to wink the eyes, as if to make them clearer, and now and then to shut them for a moment or two for rest. You need glasses if you find yourself placing the candle between your eyes and the object looked at. You then find that if you look steadily at any small thing close at hand the eye gets tired, and a dimness or watering is manifested, so as to cause indistinctness, or words on the page seem to run into each other.

It is better under these circumstances to get glasses at once. Putting it off not only does no good, but a positive and speedy injury, as the straining to see distinctly debilitates and permanently injures the nerves of sight.

Glasses are usually numbered from one up to twenty or more. First purchase number twenty, and as you observe the symptoms above named, get number eighteen, and so on. When you get to be about sixty, fourteen is usually a good number.

The glasses should be near enough to the eye to almost touch the eye-lashes. The glasses should be washed every morning in cold water, and if needed several times during the day, to remove the dust, gum and insensible perspiration from the eye, which condenses upon the inner surface of the glass, wiping with clean Irish linen, or soft, dry buckskin.

The glasses should be always carried in the case, or in a pocket in which there is nothing else; for even a newspaper can scratch them, and this rapidly impairs their clearness. The harder the glass, the more valuable the spectacles; because if scratched much, the magnifying power is diminished, and you begin to get a low number, and thus hurry the eye to old age. Brazilian pebbles, for eye-glasses, are the best, because the hardest. Cheap eye-glasses are always either soft or are carelessly matched; hence never buy a pair on the street, but from an established optician.

All devices to keep the eyes young are no more than useless, and all patents for that purpose are heartless impositions. There are some useful general rules to prevent the eyes from needing artificial aid prematurely.

Avoid reading before sunrise, after sunset, and by artificial light, or at least do those things as little as possible.

Read as little as possible before breakfast, for every part of the body is then at its weakest from the long abstinence from food; besides, as the eyes have been in perfect darkness during the long sleep, bringing them into the glare of light suddenly, and causing them to look upon the small objects, such as the letters of a word, is too sudden and too strong a contrast of use. No one ought to sew upon a dark material at night. Never use any eye wash except pure, tepid, soft water.

